# Prognostic Value of Serum Uric Acid in Patients on the Waiting List before and after Renal Transplantation

**DOI:** 10.1155/2015/375606

**Published:** 2015-01-22

**Authors:** Henrique Cotchi Simbo Muela, Jose Jayme Galvão De Lima, Luis Henrique W. Gowdak, Flávio J. de Paula, Luiz Aparecido Bortolotto

**Affiliations:** ^1^Heart Institute (InCor), Hospital das Clínicas, University of São Paulo Medical School, 05403-000 São Paulo, SP, Brazil; ^2^Faculty of Medicine, Agostinho Neto University, Luanda, Angola; ^3^Renal Transplant Unit, Urology, Hospital das Clínicas, University of São Paulo Medical School, 05403-000 São Paulo, SP, Brazil

## Abstract

*Background.* High serum uric acid (UA) is associated with increased cardiovascular (CV) risk in the general population. The impact of UA on CV events and mortality in CKD is unclear. *Objective.* To assess the relationship between UA and prognosis in hemodialysis (HD) patients before and after renal transplantation (TX). *Methods.* 1020 HD patients assessed for CV risk and followed from the time of inception until CV event, death, or TX (HD) or date of TX, CV event, death, or return to dialysis (TX). *Results.* 821 patients remained on HD while 199 underwent TX. High UA (≥428 mmol/L) was not associated with either composite CV events or mortality in HD patients. In TX patients high UA predicted an increased risk of events (*P* = 0.03, HR 1.6, and 95% CI 1.03–2.54) but not with death. In the Cox proportional model UA was no longer significantly associated with CV events. Instead, a reduced GFR (<50 mL/min) emerged as the independent risk factor for events (*P* = 0.02, HR 1.79, and % CI 1.07–3.21). *Conclusion.* In recipients of TX an increased posttransplant UA is related to higher probability of major CV events but this association probably caused concurrent reduction in GFR.

## 1. Introduction

An elevated serum uric acid (UA) is consistently associated with increased cardiovascular (CV) risk in the general population, in part because patients with hypertension, metabolic syndrome, and chronic kidney disease (CKD) frequently have elevated uric acid levels [1, 2]. Although hyperuricemia is common in patients with chronic kidney disease, the impact of uric acid on mortality and CV events remains unclear.

There are experimental and epidemiological evidences indicating that uric acid and hyperuricemia may play a role in the pathogenesis of renal and CV diseases [3]. Also, hyperuricemia after kidney transplantation seems to have an adverse effect on renal allograft survival. For instance, patients with hyperuricemia demonstrated a 5-year graft survival rate of 68.8%, compared with 83.3% in patients with normouricemia [4]. However, the Symphony study observed no significant association between uric acid concentration and worsening of renal allograft function in the first 3 years after transplantation [5].

Serum UA has also been associated with coronary artery calcification and carotid intimal thickening [6, 7]. A number of epidemiological studies have found an independent association between hyperuricemia and myocardial infarction, ischemic stroke, CV events, and all-cause and CV mortality [8, 9]. It is well known that all the above-mentioned complications are common in patients with renal disease, including those who underwent renal transplantation.

Based on these evidences, it is plausible to hypothesize an adverse effect of increased UA level on CV outcomes in patients with CKD candidates to renal transplantation. In this study we aimed to assess the relationship between baseline serum uric acid and the risk of cardiovascular events and all-cause mortality in a group of patients on the waiting list for renal transplantation evaluated before and after renal transplantation.

## 2. Methods

### 2.1. Participants and Measurements

This was a longitudinal observational study conducted in 1020 hemodialysis (HD) patients, listed to receive their first kidney graft from a deceased donor, assessed for cardiovascular risk at the Hypertension Unit, Heart Institute (InCor), University of São Paulo Medical School, Brazil, and followed from July 1999 to June 2011. Inclusion criteria were age of 18 years or older and having serum uric acid level measurement at the first visit. Patients, who had been included on the waiting list before July 1, 1999, were excluded since before that date serum UA was not measured on inception. For patients who underwent renal transplantation, we excluded those who did not have UA measurement at the first evaluation after renal transplantation.

Patients were being treated by hemodialysis, performed in 4 h sessions, three times per week with a target Kt/V of 1.3. Routine medication for patients on dialysis included aspirin, rennin-angiotensin system inhibitors, statins, and beta-blockers for all individuals independent of risk stratification. Asymptomatic hyperuricemia was not treated. Hemodialysis patients were followed from the time of placement on the waiting list until death, renal transplantation, or occurrence of CV events. Patients who underwent transplantation were followed from the time of engraftment until death, occurrence of CV event, or return to dialysis. Routine immunosuppression consisted of prednisone, mycophenolate, and tacrolimus or cyclosporine. Cardioprotective medication was maintained after transplantation, as stated above. All individuals provided a signed, written informed consent and the study was approved by the institutional ethics committee and conducted according to the Declaration of Helsinki.

### 2.2. Laboratories Parameters

Serum UA, along with routine laboratory tests, was measured on baseline in patients on the waiting list and at the first laboratory evaluation performed after transplantation. Serum UA was determined using the URCA method (Dimension Clinical Chemistry System, Siemens Healthcare Diagnosis, Newark, USA). The cut-off 428 mmol/L was used according to the reference values of our laboratory so patients were divided into two groups: those with serum acid uric level less than 428 mmol/L and those with that value or higher. For patients on the waiting list, high-sensitivity C-reactive protein levels were also determined. Increased C-reactive protein was defined by serum levels of the enzyme higher than 5 mg/L. Dyslipidemia was defined by either total cholesterol or triglycerides higher 5.18 mmol/L. For patients who underwent renal transplantation, the glomerular filtration rate (GFR) was assessed by the MDRD formula. We used the median of GFR for the whole renal transplant group (50 mL/min/1.73 m^2^) measured at the end of the follow-up, as a cut-off to define an adequate graft function. That figure is also justified because it corresponds, roughly, to a normal renal function of an individual with one functional kidney, as occurs after a successful renal transplantation.

### 2.3. Outcomes

We assessed 2 outcomes: all-cause mortality and major CV events (fatal/nonfatal), defined as sudden death, unstable angina, myocardial infarction, stroke, new-onset heart failure, and acute arterial syndrome requiring intervention. When more than one event occurred, only the first event was considered for analysis. We initially analyzed the impact of increased baseline serum UA on outcomes in patients on dialysis and in patients undergoing renal transplantation. Subanalysis was also performed separately in patients with diabetes or with increased levels of C-reactive protein. For patients who underwent renal transplantation the impact of serum UA on prognosis was also evaluated in patients with reduced (<50 mL/min/1.73 m^2^) estimated GFR.

### 2.4. Statistical Analysis

All statistical analyses were performed using the JMP statistical program (JMP for Windows, version 6.0, SAS Institute, Cary, NC, USA). Continuous variables were presented as mean and standard deviation and the categorical values as percentage. Differences between uric acid groups were tested by *χ* ^2^ test for categorical variables and Student's *t*-test for continuous variables. Survival curves were compared by Kaplan-Meier method and compared by log-rank; Cox regression model was used to assess the variables related to CV events after renal transplantation and *P* < 0.05 was considered statistically significant.

## 3. Results

### 3.1. Characteristics of Patients before and after Renal Transplantation

Table 1 shows the main clinical characteristics for patients on dialysis according to serum UA levels. Serum uric acid < 428 was observed in 838 (82%) patients whereas, in 182 (18%), serum UA was increased. For the whole dialysis population, the mean age was 54 ± 11 years and 72% were Caucasians, 59% males, 40% diabetics, 38% with associated CVD (heart failure, previous stroke, myocardial infarction, and coronary or peripheral vascular intervention), and median follow-up was 26 months. Hypertension was the most frequent risk factor affecting more than 80% of our patients. Patients with higher serum UA were younger (52.3 ± 9.9 versus 54.1 ± 11.3 years old, resp., *P* < 0.05) and dyslipidemia was more frequent in patients with elevated uric acid (41.1% versus 34.1%, resp., *P* < 0.05). All other variables were comparable between the two groups.

Table 2 shows the clinical characteristics of 199 patients that underwent renal transplantation. Serum uric acid was increased in 66 patients (33%). The main baseline characteristics for the totality of transplanted subjects were the following: age, 52 ± 11 years, 72% Caucasians, 55% males, 34% diabetics, 26% with associated CVD, and median follow-up, 19 months. There were no significant differences in clinical characteristics of transplanted patients according to their uric acid levels. However, GFR was higher in patients with serum uric acid < 428 mmol/L from the first posttransplant week onward (Table 3).

**Figure 1:**
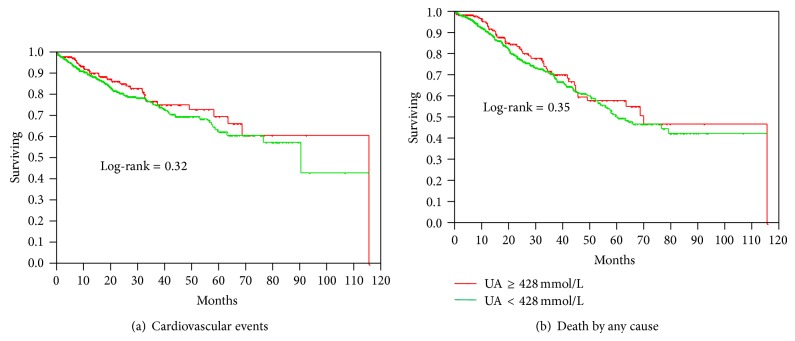
(a) and (b) Cardiovascular events and mortality in patients on the waiting list for renal transplantation according to uric acid level.

Figures 1(a) and 1(b) depict the incidence of major CV events and death by any cause in patients on dialysis with normal and increased baseline serum UA, respectively. In the dialysis group high baseline serum UA was not associated with either major CV events or all-cause mortality. In the subgroups of patients with diabetes or increased C-reactive protein an elevated UA also did not alter the incidence of events or death (data not shown).

**Figure 2:**
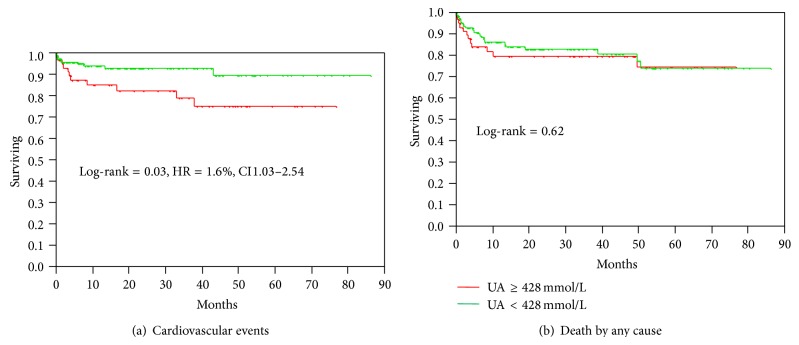
(a) and (b) Cardiovascular events and mortality in renal transplant patients according to uric acid level.

For patients who underwent renal transplantation posttransplant baseline UA ≥ 428 mmol/L was associated with increased probability of CV events (*P* = 0.03, HR 1.6, and 95% CI 1.03–2.54) but did not correlate with death (Figures 2(a) and 2(b)). In patients with estimated GFR < 50 mL/min an elevated UA had no significant impact on the incidence of events (log-rank = 0.59, HR = 1.16%, and CI 0.65–1.99) or death (log-rank = 0.61, HR = 0.90%, and CI 0.59–1.34).

Table 4 shows the multivariate analysis (Cox model) that included, as independent variables, increased serum UA, age, reduced GFR, diabetes, hypertension, and associated CV disease. Reduced GFR was the only independent predictor of major adverse CV events after renal transplantation (HR 1.79, % CI 1.07–3.21, and *P* = 0.02). Increased serum AU, age, pretransplantation diabetes, hypertension, and associated CV disease were not predictors of adverse CV events.

## 4. Discussion

In this study we examined the relationship between serum UA and cardiovascular events and all-cause mortality risk in dialysis patients before and after renal transplantation. The main finding was that higher UA (≥ 428 mmol/L) was associated with higher risk for cardiovascular events after renal transplantation. However, after adjusting for confounding factors, including GFR, that association was no longer significant. Instead, it was a reduced graft function that predicted future events. On the other hand, an increased UA did not influence the outcome of patients on dialysis or the incidence of death in allograft recipients.

Our data show that elevated serum UA was not predictor of cardiovascular events or death in dialysis patients on the waiting list for transplant. Likewise, analysis in the subgroups of diabetics and in subjects with elevated C-reactive protein showed no significant differences relative to cardiovascular events or mortality in patients with high or normal uric acid levels.

Many but not all observational studies suggest that hyperuricemia is an independent risk factor for cardiovascular mortality and/or all-cause mortality in the general population [10–12]. Culleton et al. [13], using Framingham Heart Study data, reported that an elevated serum uric acid level at baseline was not independently associated with increased risk of cardiovascular mortality. They concluded that the apparent association of serum uric acid to cardiovascular events was probably due to confounding cardiovascular risk factors.

Hyperuricemia is also common in subjects with end-stage renal disease, where it has been reported in up to 50% of subjects [14–16]. Consistent with studies in the general population, 2 studies performed in hemodialysis patients [15, 16] confirmed that hyperuricemia is also associated with an increased mortality risk in the dialysis population. It is unclear whether uric acid level is a marker for CV disease and all-cause mortality in this patient population or whether the relationship between uric acid level and mortality is independent of traditional CVD risk factors [17]. Perhaps it is more important that there is no definitive information on the effect of correction or prevention of hyperuricemia and the incidence of cardiovascular complications due to the lack of sufficient data from randomized, prospective studies on the subject.

Herein, we found that an elevated UA was associated with an increased incidence of CV events in renal transplant patients. We also observed that the proportion of patients with increased serum UA was higher in patients with reduced estimated GFR, as shown in Table 3. Since a compromised allograft function is an important predictor of CV event and death it is conceivable that the increased risk associated with higher serum UA may be explained by a compromised graft function. Indeed, in the Cox proportional model that included, besides serum UA, age, comorbidities, and GFR, an elevated serum UA was no longer significantly associated with CV events. Instead, a reduced GFR (<50 mL/min) emerged as the sole independent risk factor for major CV events. Also, in patients with reduced GFR an increased serum UA did not correlate with prognosis, contrary with that observed in the totality of patients undergoing renal transplantation. It is of interest that hyperuricemia is a common complication of cyclosporine and tacrolimus-based immunosuppression and that this alteration is attributed to reduced GFR [18, 19].

Therefore, our results favor the concept that the association of serum uric acid to cardiovascular events is probably due to confounding risk factors, especially a reduced GFR, at least in recipients of kidney transplant.

In the present investigation, pretransplant diabetes, hypertension, or concomitant cardiovascular disease was not associated with death or events in renal transplant recipients. This probably reflects the exclusion of patients with more severe comorbidities from the waiting list, leaving only individuals with mild diabetes and less advanced cardiovascular disease to be transplanted.

Why were only renal transplant recipients and not patients on dialysis affected by an increased serum UA? It was not the purpose of this investigation to answer this question. Notwithstanding, it is possible that the very high risk associated with dialysis would have superseded the relatively low impact related to an increased UA. Also, removal of UA by dialysis could have made it impossible to detect associations between increased UA and prognosis. Finally, since renal function appears to be the critical factor explaining the negative effect of UA on prognosis, in patients with no significant renal function, any association between serum UA and events would be impossible to infer.

## 5. Conclusions

Our data suggest that an increased posttransplant uric acid is related to higher probability of major CV events but suggest that this association probably caused concurrent reduction in GFR. Even so, the data may be useful to identify renal transplant patients at increased risk for cardiovascular events. More investigations are necessary to verify the impact of control in serum UA on prognosis of renal transplant recipients.

## 6. Limitations

The retrospective nature or our study may have limited the analysis of confounder factors that could influence the elevation of uric acid either before or after renal transplantation. Information regarding the use of diuretics or other drugs that could interfere with UA levels is lacking. Serum UA was measured only on inception.

## Figures and Tables

**Table 1:** Main characteristics of hemodialysis patients on the waiting list for renal transplantation.

	Total (1020)	Uric acid < 428^¥^ (838)	Uric acid ≥ 428^¥^ (182)
Age (years)	53.7 ± 11.1	54.1 ± 11.3	52.3 ± 9.9^*^
Sex (males) (%)	58.9	59.3	65.4
Race (Caucasians) (%)	71.5	71.5	71.4
Body mass index (kg/m^2^)	25.5 ± 4.8	25.2 ± 4.6	27.1 ± 4.6
Diabetes mellitus (%)	39.6	41.7	37.9
Dyslipidemia (%)	35.6	34.1	41.1^*^
Smoking (%)	24.4	25.0	24.8
Hypertension (%)	80.7	79.1	82.4
Associated CVD (%)	38.1	39.7	34.8
C-reactive protein > 5 mg/L (%)	56.5	56.0	57.9
Uric acid^¥^ (mean)	346 ± 104	310 ± 63	511 ± 89

^*¥*^mmol/L; ^*^ *P* < 0.05.

**Table 2:** Characteristics of the patients after renal transplantation.

	Total (199)	Uric acid^¥^ < 428 (133)	Uric acid^¥^ ≥ 428 (66)
Age, years (mean ± sd)	52.1 ± 10.7	52.1 ± 11.1	51.9 ± 10.1
Sex (males) (%)	55.3	51.9	62.1
Race (Caucasians) (%)	71.8	71.4	72.7
BMI^*^ (kg/m^2^) (mean ± sd)	24.8 ± 4.5	24.7 ± 4.6	25.1 ± 4.3
Diabetes mellitus (%)	33.7	33.8	33.3
Dyslipidemia (%)	35.3	34.7	36.7
Smoking (%)	17.1	17.3	16.6
Hypertension (%)	82.9	81.2	86.4
Associated CVD (%)	26.1	27.1	24.2
Uric acid^¥^ (mean ± sd)	377 ± 126	305 ± 64	521 ± 89

^*^Body mass index; ^¥^mmol/L.

**Table 3:** Mean GFR value on different time intervals after renal transplantation.

	Mean value of GFR (mL/min/1.73 m^2^)		
	Uric acid^¥^ < 428 value (*n*)	Uric acid^¥^ ≥ 428 value (*n*)	*P* value
First day	12.09 (124)	9.58 (59)	0.093
7 days	27.15 (123)	17.75 (59)	0.013
30 days	52.7 (125)	38.75 (59)	0.003
>30 days	54.85 (117)	40.72 (57)	0.0003

^*¥*^mmol/L; GFR: glomerular filtration rate.

**Table 4:** Results of Cox regression model with cardiovascular events as dependent variable in renal allograft recipients.

Variable	HR	%CI	*P*
Age at time of transplantation (≥52 yrs)	1.50	0.99–1.11	0.09
Serum uric acid (≥428 mmol/L)	1.41	0.88–2.28	0.15
GFR < 50 mL/min/1.73 m^2^	**1.79**	**1.07–3.21**	**0.02**
Diabetes mellitus	0.78	0.48–1.30	0.342
Hypertension	0.80	0.32–0.52	0.54
Cardiovascular disease	0.73	0.45–1.20	0.208

GFR: glomerular filtration rate.
